# Face processing and exploration of social signals in Prader-Willi syndrome: a genetic signature

**DOI:** 10.1186/s13023-019-1221-3

**Published:** 2019-11-15

**Authors:** Jimmy Debladis, Marion Valette, Kuzma Strenilkov, Carine Mantoulan, Denise Thuilleaux, Virginie Laurier, Catherine Molinas, Pascal Barone, Maïthé Tauber

**Affiliations:** 1 Brain & Cognition Research Center (CerCo), University of Toulouse Paul Sabatier, Toulouse, France; 2 Brain & Cognition Research Center (CerCo), CNRS, Purpan Faculty of Medicine, Toulouse, France; 3 Cerveau & Cognition, CNRS UMR 5549, Pavillon Baudot, CHU Purpan, BP 25202, 31052 Toulouse Cedex, France; 4 Prader-Willi Syndrome Reference Center, Children’s Hospital, Toulouse, France; 5 Purpan Faculty of Medicine, Purpan Hospital, Toulouse, France; 6 Marine Hospital, Hendaye, France; 7 Toulouse-Purpan Physiopathology Center, INSERM, Toulouse, France

**Keywords:** Prader-Willi syndrome, Face processing, Face exploration, Eye tracking, Social interactions, Autism spectrum disorder

## Abstract

**Background:**

Faces are critical social cues that must be perfectly processed in order to engage appropriately in everyday social interactions. In Prader-Willi Syndrome (PWS), a rare genetic disorder characterized by cognitive and behavioural difficulties including autism spectrum disorder, the literature referring to face processing is sparse. Given reports of poor social interactions in individuals with PWS, we sought to assess their face and emotion recognition skills during eyetracking recordings.

**Results:**

Compared with controls, patients with PWS performed more poorly on face/emotion recognition. We observed atypical facial exploration by patients with maternal disomy. These patients looked preferentially at the mouth region, whereas patients with a deletion and controls were more attracted to the eye region. During social scenes, the exploration became more atypical as the social content increased.

**Conclusions:**

Our comprehensive study brings new insights into the face processing of patients with PWS. Atypical facial exploration was only displayed by patients with the maternal disomy subtype, corresponding to their higher rate of autism spectrum disorder. This finding strongly argues in favor of early identification of this genetic subgroup in order to optimize care by implementing tailored interventions for each patient as soon as possible.

## Introduction

### Prader-Willi syndrome

Prader-Willi Syndrome (PWS) is a rare neurodevelopmental genetic disorder affecting the hypothalamus, characterised by endocrine dysfunctions and behaviour troubles [1]. The incidence at birth is around 1 in 20,000 [2]. The syndrome is caused by the absence of paternal gene expressions in the specific region of chromosome 15q11–13 [3]. Three different genetic subtypes have been described, arising from three distinct mechanisms: paternal microdeletion (DEL), occurring in 65% of cases; maternal uniparental disomy (UPD), occurring in 30% of cases, and corresponding to the presence of two copies of the maternal alleles in the specific region of chromosome 15; and imprinting defect, occurring in 5% of cases [4]. The ratio of DEL to non-DEL at birth currently stands at around 50%, reflecting a higher maternal age [2]. The natural history of PWS has been described and is characterized by different developmental phases from birth to adulthood [5]. At birth, infants with PWS display severe hypotonia with feeding difficulties. Around the age of 3 years, excessive weight gain begins, followed by the occurrence of obesity with hyperphagia and a satiety deficit. Thus, PWS is characterized by a specific developmental switch, from neonatal anorexia to childhood hyperphagia [4, 6–8]. Early diagnosis is now made during the first month of life, and multidisciplinary care prevents or mitigates the occurrence of severe obesity and other comorbidities.

### Cognitive abilities in PWS

In addition to the clinical features described above, related to a specific hypothalamic dysfunction [1, 9], cognitive impairments are also present. Individuals with PWS usually have mild-to-moderate intellectual disability (ID) with a mean intellectual quotient (IQ) of around 65–70 [10]. They display learning difficulties and poor working memory capacity when performing tasks that simultaneously require different cognitive abilities [11]. Compared with other genetic syndromes with mild ID, patients with PWS have a higher rate of additional behavioural problems. These include temper tantrums, impulsivity, mood fluctuations, stubbornness and aggression, as well as a range of repetitive behaviours such as skin picking, repetitive speech, and obsessive and ritualistic behaviours [10, 12, 13]. Indeed, it is now established that individuals with PWS display behavioural features of autism spectrum disorder (ASD) [14] with deficits in aspects of theory of mind [15], social abilities and understanding of emotions resulting in social weakness, social interaction problems and poor relationships with others [16, 17]. This, together with the hyperphagia explains their poor and complex socialization. While the hypothalamus has been the major area of focus in PWS, other brain regions, that belong to the social brain network (see Mantoulan et al. [18], Tauber & Payoux et al. unpublished), are likely playing an important role in the pathology, although their function and role are still understudied.

### Difference between UPD and DEL genotypes

In the past few years, research on PWS has differentiated between the two genetic PWS subtypes mainly in terms of physical and behavioral characteristics. DEL patients are often more atypical in their clinical physical features than UPD patients [19]. Although the genetic subtypes have similar full-scale IQ scores, performance IQ scores are higher for DEL, while verbal IQ scores are higher for UPD. Differences in learning skills (mathematical, word meaning, social understanding) and factual social comprehension have also been identified with UPD patients performing better [20].

In addition, patients with the DEL versus UPD subtype perform differently in visuospatial tasks [21] and in visuomotor integration [22], suggesting that visual processing is more efficient in DEL than UPD.

Moreover, UPD patients have higher comorbidity rates for ASD-like features than DEL patients [14, 23]. The prevalence of ASD features is about 45% for UPD patients, and close to 20% for the DEL patients [24]. The former have a higher risk of developing psychiatric problems, such as affective disorder, ritualistic behavior, and psychotic disorders [10, 25].

### Social signal processing in PWS

Successfully detecting and processing social signals is vital for interacting with our social world. The brain pathways implicated in face and voice processing have been well studied, and involve a large number of connecting structures belonging to the *social brain* (for more details about brain structure, see [26]).

Given the ASD comorbidity seen in PWS, and the abundant literature documenting impaired social processing and social attention in ASD populations [27], we were interested in assessing and characterizing social signal processing in PWS, adopting a comprehensive approach. Neuropsychological studies corroborate the finding that patients with PWS exhibit cognitive impairment in social processing tasks [14, 28, 29]. We had previously studied the ability of patients with PWS to discriminate human voices from environmental sounds during a forced-choice task. We showed that their overall performances were poorer than those of controls, meaning that individuals with PWS have a human voice processing deficit [30]. We then looked at another important social skill, namely face processing, and found that the literature in PWS is sparse and contradictory [21, 28, 29]. While the study of Key et al [29] found that only UPD patients didn’t present typical ERP face response, the study of Halit et al [28] reported an overall typical face scalp distribution in PW group and behavioural measures in the normal range. On the opposite, the study of Feldman et al [21] reported a behavioural deficit in PWS during a face discrimination task, while both genetic subtypes being similarly affected. Altogether these limited number of studies present several aspects of disagreement while pointing toward the fact that patients with PWS display impaired face processing skills that need further investigation. In particular, they have difficulty recognizing facial expressions (Ekman faces test), and their emotion discrimination is correlated with socialization measures [31].

### Aims of the present study

The aims of the present study were to i) confirm and complement previous findings on social cues in PWS, ii) decipher face processing skills in PWS, by analyzing oculomotor strategies, and iii) compare genetic subtypes on social processing. Our overall goal was to understand the deficits in social skills in order to offer effective rehabilitation for these patients, their families and carers, thus improving their daily life and socialization.

## Results

### Performance on face/emotion discrimination task

**Fig. 1:**
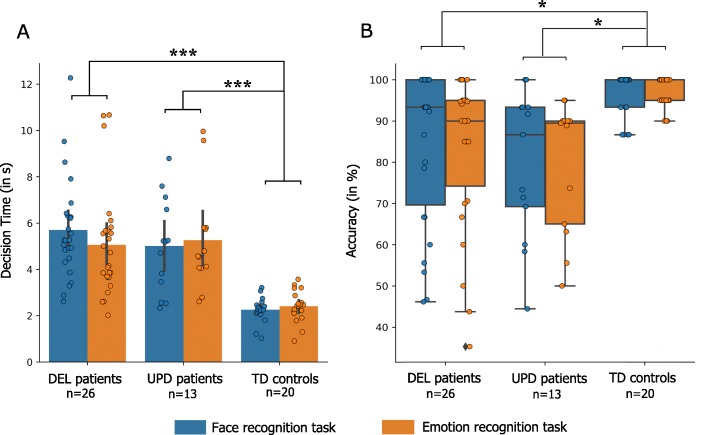
Overall performances during the face and emotion recognition tasks. **a**: decision times (RTs) during the face/emotion discrimination task. Mean RTs are presented with standard deviation and population’s distribution. The asterisks represent statistical differences between TD controls and patients with DEL or UPD. **b**: Median accuracy and population’s distribution obtained for the face/emotion discrimination tasks. The asterisks represent significant differences between the mean percentage of performances between the TD controls and patients with DEL or UPD. Patients with PWS were slower at making decisions and performed more poorly on the face and emotion recognition tasks than the TD population. We did not find any differences between the two tasks or between the two genetic populations

Only one patient failed to perform the tasks. In the analysis of reaction times, significant effect of group (*p* < 0.001) was found. In both tasks, patients with PWS performed twice as slowly as controls (DEL: 5.4 s; UPD: 5.2 s; Controls: 2.3 s). We found no differences in response times between the two PWS genotype subtypes (Fig. 1.A), either for face (*p* = 0.53) or for emotion recognition (*p* = 0.95).

Controls’ performances were almost at ceiling effect in both tasks (Mean: face: 96.3%, Median: 100%; Mean: emotion: 96.5%, Median: 95%; Fig. 1.B). In the analysis of performances, significant effect of group (*p* < 0.001) was found. Compared with controls, PWS had a small but significant deficit in face and emotion recognition (Mean: UPD: 79.7% *p* = 0.03; DEL: 84.1% *p* = 0.02, Median: UPD: 89.2%, DEL: 93.3%, Fig. 1.B). No differences were observed regarding type of stimulus for each population (face vs. emotion) nor between the subtypes of patients (face *p* = 0.62, emotion *p* = 0.74), neither according to the gender. However, out of the 22 patients who made at least one error (only 7 in TD group), 10 are DEL patients (corresponding to 38% of the DEL population) and 12 are UPD patients (corresponding 92% of UPD population). These results emphasis the fact that in spite of an absence of difference compared to DEL patients at the performance level, the UPD population is probably more affected in their deficit in processing visual face information.

Lastly, concerning the type of emotion to categorize, we also found that, in the PWS population, the happiness is the easiest emotion to discriminate (26% of the errors) and the sadness and the fear are equally badly recognized (respectively 36 and 37% of errors). In the TD population, the fear represents almost all the errors made by the subjects (91%).

We used a Bayesian estimation of the drift diffusion model (DDM) to analyze individual performances [32]. This makes it possible to assess how much information individuals need to make a decision, thus separating decision criteria from nondecision processes (e.g., perceptual and motor aspects). This model revealed that, compared with controls, individuals with PWS had a higher threshold (PW = 5.76 vs TD = 3.5) associated with a lower drift rate (PW = 0.62 vs TD = 1.5). Regarding genetic subtype, we found that the DEL patients had a higher threshold than UPD patients. Moreover, patients with the UPD subtype exhibited a bias (z = 0.57) toward the correct response, unlike those with the DEL subtype (z = 0.49, i.e., chance level). Nevertheless, we did not find any difference in drift rate (v) between the DEL and UPD subtypes. Finally, motor responses were lower in the UPD population than in the DEL population (more details in Supplementary data). All these analyses revealed that the deficit of PWS patients originated from a deficit in the process of decision making especially concerning UPD subtype.

### Oculomotor exploration

We excluded 12 patients from the oculomotor analysis because the eye movement recordings were not accurate enough (fewer than 50% of total frames recorded). All the details concerning the exclusion criteria used in the analysis are provided in the *Materials and Methods* section.

#### Oculomotor behaviour for response selection

We observed differences between patients with PWS and controls on the amount of time spent exploring each face, especially when we distinguished between PWS subtypes. TD Controls and DEL patients spent significantly less time exploring the distractor (26.1 and 27.9%) than the target face (34.1 and 39% respectively *p* < 0.001 and *p* = 0.002) and sample face (38.7 and 37.2% respectively *p* < 0.001 and *p* < 0.001). Owing to the variability in UPD behaviors, the statistical analysis revealed only a difference in the distribution of fixations for this subtype whose spent significantly less time exploring the distractor than the target face (27.7 and 34.5%, *p* = 0.01).

**Fig. 2:**
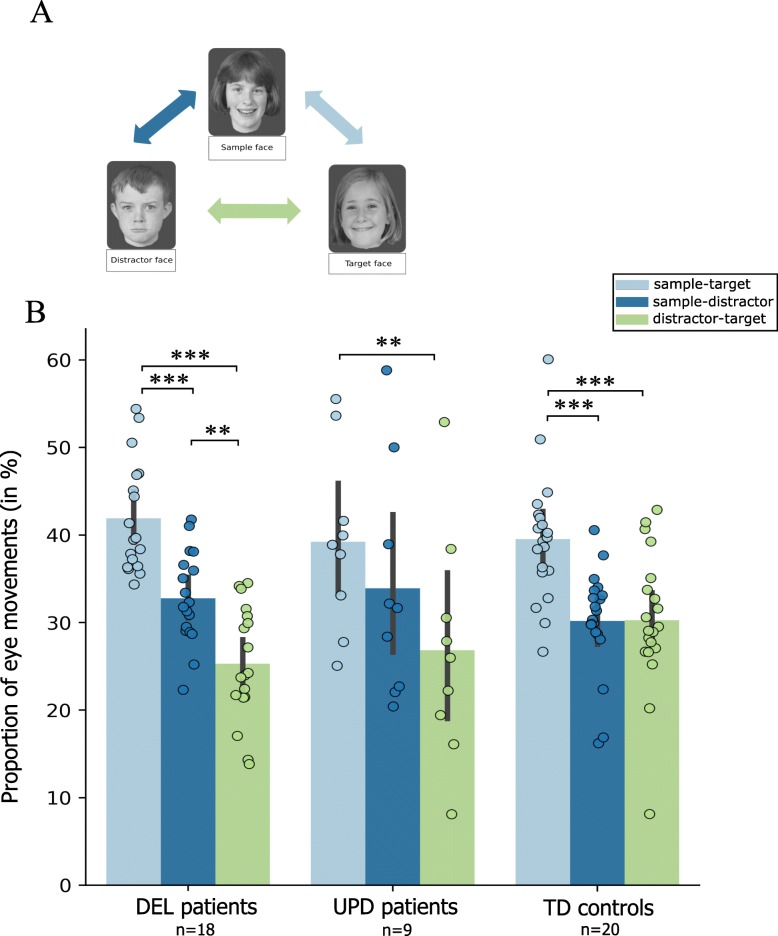
Exploration strategy expressed as the saccadic rate. Quantitative distribution of saccades between all the faces, expressed as means and standard deviation. On each barplot, the distribution of individual values is demonstrated. In our study, a saccade was an eye movement between two faces, regardless of direction. The TD population and patients with DEL preferentially made saccades between the sample and target faces. Furthermore, whereas the TD population equally distributed their saccades between the other faces, the patients with DEL made more saccades between the sample and distractor faces than between the distractor and target faces. For individuals with UPD, the high level of variability meant that the relative numbers of saccades between the three faces were statistically similar

To complement the fixation time data, we analyzed the saccadic behavior associated with the exploration of the three faces. PWS subjects generally made fewer saccades than controls (one eye movement every 662 ms for PWS vs. every 510 ms for controls; data not shown). Moreover, controls and DEL made significantly more saccades between the sample and the target face (39.6 and 41.9%; Fig. 2) than sample-distractor or distractor-target faces. Lastly, owing to the variability in the eye movements of UPD, only one proportion of saccades was statistically significant, between sample/target and sample/distractor faces (respectively 39.3% vs 26.8%, *p* = 0.002).

#### In-face exploration

**Fig. 3:**
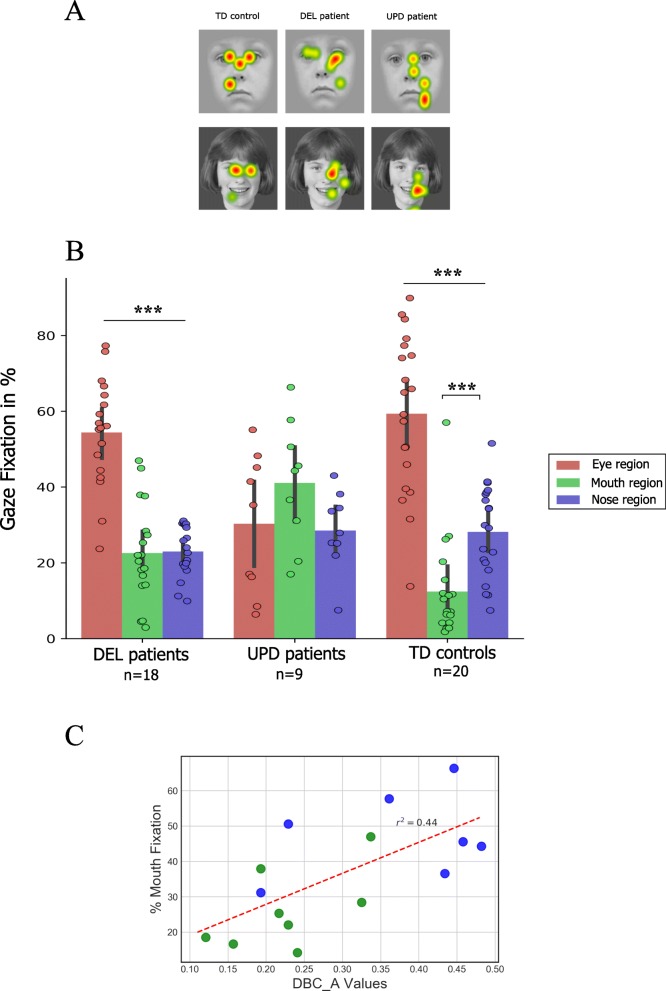
Face exploration pattern using AOI analysis. **a**: Examples of heat maps of eye fixations for three typical participants in each group. The redder the color, the longer the fixation time. **b**: AOI (eye, mouth, and nose) gaze distribution for all the faces displayed on the screen. The asterisk represents a statistical difference between two mean percentages of gaze fixations for two AOIs. For the TD controls and DEL patients, the eye region is the most gazed area compared to the 2 others face areas. For the population with UPD, no statistical differences between AOIs were found. Moreover, compared to TD and DEL patients, UPD patients looked longer at the mouth region. **c**: Scatter plot of the DBC_A scores as a function of the percentages of mouth fixations for each participant with PWS. The green dots represent the patients with the DEL subtype, and the blue dots the patients with the UPD subtype. The regression line is represented by the dashed red line

A precise quantitative analysis of the fixation times for all three faces revealed that controls and DEL were both most attracted to the eye region (59.4 and 54.4%; Fig. 3.B). Whereas controls explored the mouth region less (12.4%), DEL explored the mouth and nose regions to the same extent (22.6 and 23%; Fig. 3.B). A clear difference emerged when we compared the two genetic subtypes. UPD looked significantly longer at the mouth region than either controls or DEL did. Compared to TD controls, UPD patients present a significant higher fixation duration directed toward the mouth region (12.4% vs. 41.1% respectively, *p* < 0.001) with the distribution of UPD patients being over the individual values obtained for TD individuals. In addition, the intergroup comparison revealed also that DEL fixation values are significantly lower to that obtained in the UPD population (*p* < 0.001) but are not significantly different from TD values (*p* = 0.69). Such results suggests that the DEL population appears to be intermediate between the normal values and the abnormal behavior of the UPD genetic subtype.

Importantly, a separate analysis confirmed that faces were similarly explored during the two discrimination tasks (face and emotion) for saccades behavior and gaze distribution in all tested populations. Moreover, except on the mouth region in UPD group (Face: 30%, Emotion: 52%, *p* = 0.001) and on the eye region in DEL group (Face: 60.8%, Emotion: 48.1%, *p* = 0.04, see in supplementary data), the percentage of fixation on the AOIs defined in this study were similar between the two discrimination tasks.

Lastly, we search for a gender effect on the face exploration and report no effect of gender on these parameters.

### Correlation analysis

In the correlation analysis, all the PWS patients were globally included without genetic distinction in order to take into consideration the individual clinical assessment. The clinical DBC assessment was available in a restricted set of 15 patients. We found no correlations between IQ and face/emotion recognition performances (Spearman’s rho = 0.33, *p* = 0.09). The one significant correlation was between the overall DBC_A score and total fixations of the mouth region (rho = 0.61, *p* < 0.05; Fig. 3.C). Importantly, we report no other correlation, even between behavioral measures and eye/mouth fixation times nor with hyperphagic scores.

### Videos analysis

**Fig. 4:**
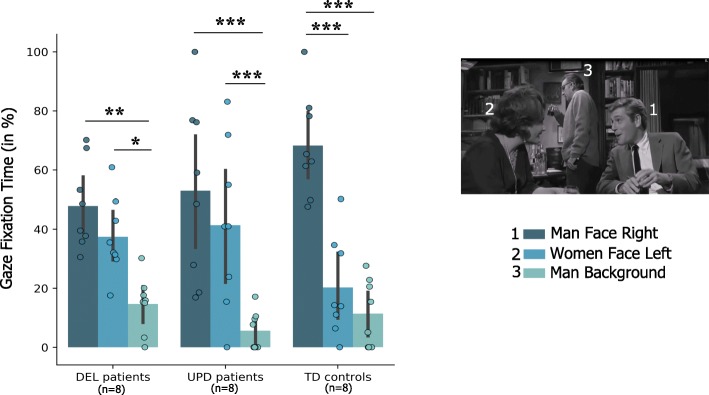
Visual exploration during video presentations. Mean gaze fixation time during the first part of the first sequence, expressed as a percentage of the total fixation time and represented as standard deviation and individual distribution. Patients with PWS (DEL plus UPD) were equally focused on the speaker and the woman listening on the left of the scene. TD controls gazed predominately at the man speaking on the right of the scene. The asterisk represents a statistical difference between the mean gaze fixation percentages for two AOIs

In the first part of the first sequence, controls were more attracted to the speaker, and fixations on the other two characters were equally low. Patients with PWS exhibited a radically different gaze pattern, as they looked at the woman listening to the conversation as much as the speaker, dividing their attention between the two faces in the foreground. The man in the background was ignored to the same extent by all three populations (Fig. 4).

In the second part of the first sequence, results showed that patients with PWS had the same exploratory gaze behavior as controls. Participants fixated the woman speaking and ignored the rest of the scene. For the other two sequences, no differences the gaze behaviors were found (Additional file 1).

## Discussion

Participants with PWS exhibited a general face/emotion recognition deficit, despite the low difficulty of the task. We also demonstrated for the first time a significant difference in face exploration between the two main genetic subtypes. In addition, during the video presentation, all individuals with PWS demonstrated atypical exploration, but only when the social content increased. Taken together, these deficits may contribute to the poor social skills observed in patients with PWS that prevent socialization, despite only mild ID.

Patients with PWS performed the tasks twice as slowly as controls, possibly owing to the general cognitive processing slowdown revealed by the DDM (see below).

We demonstrated a deficit in face and emotion recognition in line with previous studies of unfamiliar face recognition [20, 28] using an adapted version of the Benton Facial Recognition Task, which showed that patients with PWS have impaired unfamiliar face association. As in our study, they did not observe any differences between genotypes. The precise recognition of the emotions is a crucial point for social interactions as our exchange with others are modulated and adjusted according to the correct identification of their intention. One can hypothesize that if patients were at least partly impaired in emotion recognition, their behavior will not be adapted leading to, self-restraint and reducing the search for social interactions.

Surprisingly, patients displayed the same accuracy in both the face and emotion recognition tasks. In general, emotion recognition is more difficult than face recognition, but in the present task, patients did not have to recognize the emotion per se. They probably developed recognition strategies based on local face features, sufficient to process facial emotions, just as patients with prosopagnosia do [33]-a hypothesis that needs to be confirmed, given the difficulty individuals with PWS have recognizing and/or understanding emotions in real life.

To our knowledge, this is the first study to have used eye tracking to clarify atypical visual exploration by documenting the strategies used to scan faces displayed on a screen. By analyzing the patterns of saccades between the faces, and the amount of time spent fixating each face, we showed that only UPD patients had an abnormal profile*.* The difference in the ocular strategies for face and/or emotion recognition between DEL and UPD subtypes was primarily related to the variability observed in the UPD subgroup due, in part, to a reduced number of patients.

Interestingly, compared with controls, DEL and UPD patients made more saccades between the sample and distractor face. This is further evidence of the difficulty caused by poor working memory skills in PWS [34–36] which makes it harder to store large numbers of facial features. The patients with PWS may have had to compare more individual features, increasing the number of saccades. Another possible explanation is that patients with PWS needed to extract solid information to perform the face and emotion recognition tasks, resulting in an increased proportion of saccades. This hypothesis was confirmed by the Bayesian estimation of the DDM (see [32]), which revealed that the threshold was higher, in addition to a slower drift rate. Taken together, it suggests that in order to make face discrimination decisions, patients with PWS had to encode more information, resulting in far slower and less accurate cognitive processing, compared with controls.

In a visual scene, attention is usually automatically shifted to a face, even when this face is not the most salient cue in the scene [37] or other parts of the body are also visible. We automatically extract information about people’s intentions or emotional states by looking at their eyes [38]. A large number of eyetracking studies have demonstrated that controls predominately look at the eye region of the face, unlike patients with ASD [39]. In our study, the AOI analysis revealed that the PWS population differed in terms of gaze preferences. Whereas the DEL patients behaved like the TD population, UPD patients preferred to look at the mouth region. If they shift their gaze to the mouth region, patients with PWS potentially encounter difficulty understanding and deciphering social cues or extracting all the information needed for appropriate everyday social exchanges. Moreover, we found an increase in the percentage of fixation on the mouth area for patients with PWS during the emotion discrimination task. This suggests that under certain conditions patients will automatically focus their gaze towards the mouth area to recognize specific emotions.

According to the “eye contact effect” [40], which is characterized by an automatic gaze shift to the eyes in a social interaction, the development of typical social daily interactions and the decoding of the emotion portrayed by a face are tuned by our affinity to the eyes. One can consider that the weakness in establishment of pair social interaction and the poor emotion recognition and comprehension in UPD patients could be related to their atypical face scanning.

Some authors have reported that, compared with a typical matched population, adults with ASD pay reduced attention to social stimuli [41, 42], display a deficit in face processing [43] and show atypical social viewing during dynamic presentations, avoiding the eye region. Atypical visual exploration has also been observed during natural presentations, with decreased attention paid to social visual stimuli by individuals with ASD [44]. In our study, patients with the UPD subtype had a deficit in face processing, with aberrant gaze visual exploration, reminiscent of previous findings in ASD research.

We did not report any correlation between eyes fixation time and face/emotion discrimination performances. Such lack of correlation could be due to a ceiling effect. Indeed, the task was quite easy and such high performance level, while being significantly lower to that obtained in controls, precludes to provide robust correlation with the multiple aspects of gaze exploration. Moreover, as discussed below, the patients with PWS could use an adaptive strategy based on a more local approach to indicate which faces were similar or presented the same emotion. To recognize some emotions as fear or disgust, conveyed by the upper half of the face [45], or during a task involving theory of mind abilities, the decrease in eye fixation might be more problematic for UPD patients than during our face discrimination task.

Interestingly, we found that the patients with PWS were not systematically impaired in gaze fixation during the free viewing video task. When there was a dyadic episode (i.e., only two actors), patients and controls spent similar amounts of time spent looking at the social elements of the scene, but when there were three actors, gaze exploration became atypical. This behavior was again comparable to what has been reported in ASD [46, 47]. We concluded that in PWS, as in ASD, increasing the social content leads to social impairments. In our clinical practice, we find that patients with PWS communicate better when there are only a few people present. When there are more than two people, they adopt an ASD-like behavior, avoiding eye contact.

In patients with ASD, the amount of time spent fixating the mouth region correlates with the severity of their social deficit. Thus, shifting the gaze to the mouth region signals social processing abnormalities in PWS. This finding reinforces the idea that patients with PWS (especially the UPD subtype) should be regarded as *autistic* patients and exhibit a higher ratio of autistic comorbidities and psychopathological illness [48]. Jones and colleagues reported that decreased attention to the eye region in children with ASD is a predictor of social deficits in adulthood ((53, 54)). However, as the fixations on AOIs were dependent on each other in our study, mouth fixations were also a good indicator of social impairment in PWS, and could be used in routine practice for early detection and early rehabilitation.

Taking in consideration the limited number of UPD patients, our study still revealed a distinction between the two genetic populations concerning the face exploration. Although they had identical performances, patients with UPD exhibited ASD-like behaviors, while those with DEL explored the faces in the same way as controls*.* Furthermore, increased gaze fixations on the mouth region correlated with better language capacities [49, 50] which are important for acquiring better language skills later on [51]. The higher verbal IQ in the UPD genetic subtype could be related to this finding.

The distinction between UPD and DEL patients has also been documented during EEG recordings of face processing components. In a typical population, the N170 is modulated during inverted face presentation-a mechanism also present in the DEL subtype. However, patients with the UPD subtype display an altered N170 response, related to findings in ASD [29]. Furthermore, an EEG study aimed at evaluating incidental memory in a PWS population using repeated faces, found that presenting a new face failed to modulate the ERP responses of patients with the UPD subtype [52]. This absence of modulation, similar to that observed in patients with ASD [53] was interpreted as a deficit in attributing motivation values to social information such as faces-a deficit that could be related to the similarity of UPD symptomatology to that reported in ASD. Another study argued that patients with the UPD subtype have poorer visuospatial abilities than those with the DEL subtype-a deficit implicating the ventral temporal cortex, which is also involved in face identification [21]. More generally, a dysfunction of the visual ventral stream, part of the social brain [54], could explain the deficit in face processing reported in the UPD subtype.

Finally, using a voice discrimination task, we confirmed in a previous study [30] that patients with PWS and, more precisely, the UPD subtype, have difficulty distinguishing voices from environmental sounds. Voices, like faces, constitute an important social cue. Therefore, when coupled with atypical face scanning, a deficit in processing voices amplifies social misunderstanding in the PWS population. The voice processing deficit reinforces the idea that patients with the UPD subtype have a general social deficit that is more pronounced than in the DEL subtype.

## Conclusions and limitations

To conclude, the present study yielded fresh insights into face processing in PWS, showing that patients have impaired face and emotion recognition-a deficit that is partly related to atypical eye/face exploration. It also revealed a difference between the two main genetic subtypes, suggesting that patients with UPD behave like the ASD population. It is therefore crucial to distinguish between the two subtypes as early as possible, so that rehabilitation has the maximum impact on social communication abilities. This, of course, presupposes early determination of the genetic subtype as part of routine diagnosis. Finally, as PWS is detectable a few days after birth, it could be regarded as a good developmental model for studying social impairments in ASD.

The principal limitation of this study concern the relatively low number of patients with UPD sub-type included. For eye-tracking analysis, the analyzed population of a restricted set of 9 subjects reduced the statistical power. It could be important to confirm our analysis on a larger cohort of patients to validate our novel findings regarding face exploration in PWS.

## Methods

### Participants

The adult patients (mean age 28 years) with PWS who were included in this study were assessed either at Hendaye Marine Hospital (*n* = 11), a dedicated rehabilitation center for adults with PWS, or during a consultation at the reference center for PWS at Toulouse University Hospital (*n* = 28), in which case the experimental testing was performed at the Brain and Cognition Research Center (CerCo) located in the hospital. The total sample, comprising 15 males and 24 females with PWS (Table 1), was compared with 20 typically developing controls matched for age and sex. The study was approved by the ethics committees of Toulouse University Hospital (CHU 13687203; National EudraCT 201,300,437–33) and the experiment was conducted in accordance with the Declaration of Helsinki (2013). Prior to their inclusion in the study, all participants (and/or their legal guardians) gave their full written informed consent.

**Table 1:** Clinical and genetic characteristics of the patients with Prader-Willi syndrome (PWS) and typically developing (TD) controls. We tested 39 patients with PWS (26 with a deletion and 13 with a disomy) and 20 age- and sex-matched controls. Means and standard deviations are provided for age, full-scale IQ, and the DBC_A score. The two main genetic subtypes were not statistically different (controlled by Wilcoxon test)

Population	Age	Sex		Total	FSIQ	DBC
	Mean (SD)	M	F		Mean (SD)	
PWS	28,02 (8,03)	15	24	39	57,03 (10,02)	0,24 (0,12)
TD	24,1 (2,61)	8	12	20		
DEL	28,1 (7,4)	15	11	26	57,9 (10,37)	0,20 (0,14)
UPD	28,4 (9,7)	4	9	13	55,4 (10,15)	0,29 (0,09)

### Genetic evaluation and clinical assessment

The genetic determination was perform for each participant prior to the inclusion in the study. For deletion a 15q11q12 QMPSF assay was used (Quantitative Multiplex PCR of short fluorescent Fragment). In some patients a Fluorescence in-situ hybridisation (FISH) analysis has been performed to detect ch15q11-q13 deletions. If the QMPSF is negative, we searched for the presence of a maternal disomy using a DNA polymorphism analysis completed on the proband and the parents. For the methylation abnormalities, a methyl specific PCR test at the SNURF-SNRPN locus was accomplished. All the deletion genotyping were performed on the reference centre at Toulouse.

The Developmental Checklist for Adults (DBC_A), a questionnaire completed by parents or caregivers for the assessment of behavioral and emotional problems in adults with developmental disorders and ID, is routinely used for patients with PWS [55]. The total questionnaire contains 107 items rated in a three points scale ranging from 0 (not true), 1 (somewhat or sometimes true) and 2 (very true always true). It is divided into six different categories: disruptive, communication and anxiety disturbances, antisocial, self-absorbed, depressive, and social relating. The raw scores have been computed and normalized by the number of items in each category. For the correlation, we have used the normalized total score ranges in our population from 0.06 to 0.59.

### Face processing protocols

The tasks were adapted to the patients’ cognitive deficits. The instructions were given by the experimenter immediately prior to each experiment, to maximize participants’ comprehension of the task. The experiment began when the example was fully understood and had been successfully performed.

#### Face/emotion discrimination task

**Fig. 5:**
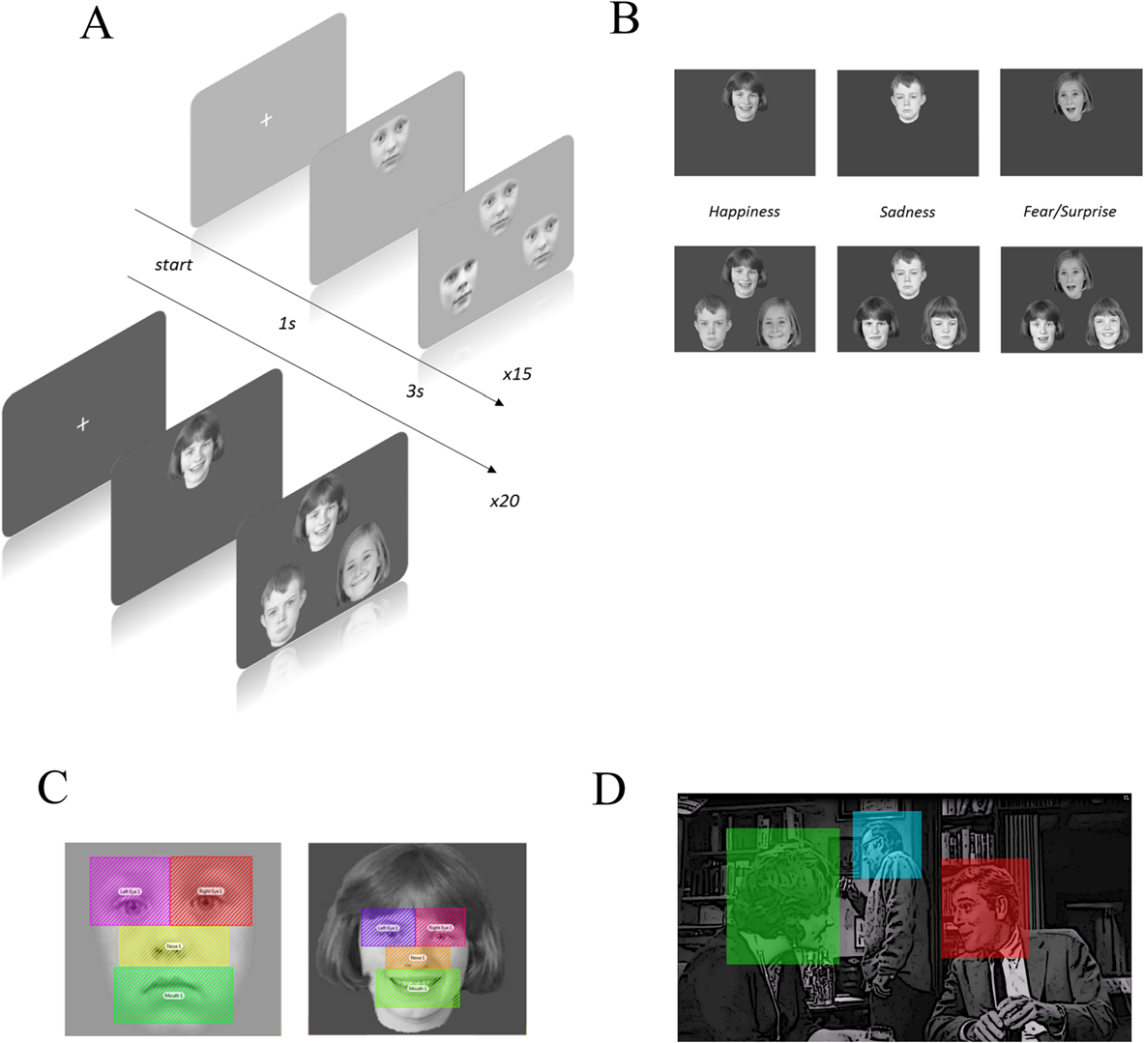
Description of the methodological aspects of this study. **a**: Design of the two experiments performed by participants: face discrimination task (top) and emotion discrimination task (bottom). Face or Emotion tasks are presented in a block of respectively 15 and 20 trials. Taking into account the patients’ well-known fatigability, the task did not last more than 5 min. For the face recognition task, all the external features (hair, body) were deleted, so only the internal facial features could be used for recognition. **b**: Examples of the areas of interest (AOIs) used in the analysis of the eye-tracking data. C: Schema of the scene from the movie that illustrates the AOIs determined in the first part of the first sequence: the face of the speaker on the right-hand side, the face of the woman on the left-hand side, and the face of the man in the background. The screen was also defined as an AOI (not shown in this figure)

Participants had to recognize either two similar faces or two faces displaying the same emotion. First, the sample face was displayed on its own in the center of the upper part of the screen for 2 s. It was then joined by the target face (either the same as the sample face, or showing the same emotion) and a distractor face in the lower half of the screen. The instruction was to identify the two identical faces (with or without different profiles) or the faces displaying the same emotion. To do this, participants gave their responses by pressing right or left adapted keyboard buttons corresponding to the position of the face. To reduce task difficulty, we did not impose any time limit. Altogether, 15 (face) and 20 (emotion) different associations and faces were presented in a pseudorandom order (see Fig. 5.A). For the emotion task, we used three different basic emotions: happiness, sadness and fear (Fig. 5.B). For face discrimination task, we have used 5 individual faces under 2 different conditions: front and profile. Altogether, the 15 presentations were unique but composed by one of the 5 individual faces chosen (front/front, front/profile and profile/profile) which could be repeated.

For each participant, we have calculated an average accuracy score expressed in percent and decision time expressed in seconds.

### Eyetracking recording

While the patients performed the face discrimination tasks, their eye movements were recorded to analyze the oculomotor strategies they implemented to perform the task. We used two different eyetrackers: a portable Mirametrix at Hendaye Hospital, and a Tobii X2–60 at CerCo. The results were separately analyzed, compared and then pooled, as no differences were observed between the two devices.

For each face (sample, target or distractor), we defined four different areas of interest (AOIs): eyes, nose, mouth, and one located outside the faces (see Fig. 5.C). We choose to design square AOI because of definition and accuracy of the eye tracker gaze location. It’s also for this reason that we have included eyebrow in the eye area.

In addition to the AOI analysis, we studied gaze distribution, as well as saccades between the three faces displayed on the screen. We calculated gaze distribution by counting the fixations on each face (sample, target and distractor), divided by the total number of fixations on all three faces. Finally, we considered eye movements (but not their direction) between faces, to determine the number of saccades.

### Exclusion criteria before ET analysis

For eye tracking analysis, we have excluded 12 patients for different reasons: the first one is when a patient was not able to perform the calibration phase of the eye tracker. In this case, we have considered that the position of the eyes was not accurate enough to represent the exact location of the gaze exploration. Moreover, a large number of patients wore glasses and in these cases no reflection from the cornea is accessible to the eye tracker detector and consequently no gaze point can be recorded. Lastly, we considered that when below 50% of the recorded gaze points (named frames), the pattern of the gaze exploration was not accurate and robust enough. Below this threshold, the gaze points in a specific area could be obtained by chance. To be more conservative in our analysis, we have defined a fixation as a succession of 3 recorded points.

The general descriptive table (Table 1) comprises these excluded patients. Among these 12 patients, 4 were UPD patients and 8 were DEL patients.

### Movies

In addition, the last participants were instructed to watch three short sequences of the movie *Who’s Afraid of Virginia Woolf*?, which had already been used by Klin and colleagues to test individuals with ASD [56] (DEL *n* = 8, UPD n = 8 and TD n = 8). Participants were instructed to explore the video as they wished. To ensure that they were attentive, we asked them a question about the sequence they had just seen at the end of each video clip. For each movie, we performed a basic AOI analysis for the key socially relevant parts of the scene (Fig. 5.D).

The movie was divided into 2 distinct parts based on the content of the sequence in terms of actor’s interactions. The first sequence is of 35 s of duration and is marked by a change of plan: in the first part lasting 15 s, the characters interact and are talking to each other in a wide shot plan showing the 3 characters. Then in the second part of 20 s of duration, only two characters are present close up facing the camera.

### Statistical analysis

As our dataset was not normally distributed, we used a linear mixed-effects model using LME4 package on R. We have analyzed reaction time and accuracy on the one hand, and on the other hand, we have analyzed all the percentages of gaze fixation (saccade, gaze distribution between the faces and AOIs). For each parameter, we used as Group Factor (TD controls, DEL patients and UPD patients), and condition factor (face or emotion) and Group: Stimulation interaction. ANOVA are performed to estimate the models. After, post-hoc test are performed to adjust *p* values for multiple comparison (pairwise). In the graph, we have used bootstrap method for calculate 95% confidence intervals. We performed a correlation analysis based on the oculomotor behavior and the DBC_A values obtained for 15 patients, using Spearman’s rank correlation coefficient.

For clarity’s sake, the face and emotion recognition results are grouped together, as we found no important statistical difference between them.

## Supplementary information

**Additional file 1.** Results of the Bayesian estimation of the drift diffusion model employed to decision making. Details of average values for each eye-tracking parameters during face and emotion discrimination tasks and between all the tested populations. Lastly, tables of the three sequences of the movie used in this study are provided.

## Data Availability

The datasets used and/or analyzed during the current study are available from the corresponding author on reasonable request.
