# Supplementary material for: Face processing and exploration of social signals in Prader-Willi syndrome: a genetic signature
Source: Orphanet J Rare Dis. 2019 Nov 15;14:262. doi: 10.1186/s13023-019-1221-3 (PMC6858697; doi:10.1186/s13023-019-1221-3)
Supplement: Supplementary file 1 — Additional file 1. Results of the Bayesian estimation of the drift diffusion model employed to decision making. Details of average values for each eye-tracking parameters during face and emotion discrimination tasks and between all the tested populations. Lastly, tables of the three sequences of the movie used in this study are provided. [file 13023_2019_1221_MOESM1_ESM.docx]

**Additional file**

**Hierarchical Drift Diffusion Model (HDDM)**

The hierarchical drift diffusion model (HDDM) represents the Bayesian estimation of the classic drift diffusion model proposed by Ratcliff et al. in 1978 ^1^. The DDM is a sequential sampling model that correlates response accuracy with reaction times (RTs) from simple two-alternative forced-choice decision-making tasks. It postulates that each decision can be modulated by the accumulation of noisy information over time. Occurrences accumulate until they reach a threshold, when the individual takes a decision. Each decision is represented by an upper and a lower boundary that have to be crossed in order to initiate the corresponding response. A schema of the theoretical functioning of the HDDM, adapted from ^2^, is provided in Figure S1.A.

Adopting the Bayesian approach to the DDM can therefore shed light on the cognitive and psychological processing behind decision making, based solely on RT distribution for the two response choices. With this model, the behavioral data can be divided according to four parameters: threshold (a), drift rate (v) for the accumulation speed, nondecision time (t) associated with stimulus perception, and response execution and a priori bias (z).

We used the Monte Carlo and Markov chains (MCMC) method to estimate posteriors based on our data. We performed 20,000 iterations. As the model takes time to *burn in*, we discarded the first 5000 items, and only saved every fifth sample. This method yielded 3000 posterior values that were normally distributed.

When using MCMC sampling, it is crucial to make sure that the chains have converged, to ensure that the samples are drawn from the posterior distribution. We confirmed our model using the posterior plots available in Python software (PyMC). From this simulated population, we could calculate the mean and 95% confidence interval for each parameter.

Using this approach, we demonstrated that our PWS population differed in cognitive aspects from the TD population (Fig. S1.C). Patients needed more information (a = 5.76) and had a lower integration speed (v = 0.62) than controls. They were not biased towards the correct face/emotion (z = 0.39), and displayed slow motor execution (t = 0.84).

Regarding the genetic subtypes, we found that the DEL patients had a higher threshold than UPD patients, meaning that the former need more information before giving their response than the latter. Moreover, patients with the UPD subtype exhibited a bias (z = 0.57) toward the correct response, unlike those with the DEL subtype (z = 0.49, i.e., chance level). Nevertheless, we did not find any difference in drift rate (v) between the DEL and UPD subtypes. Finally, motor responses were lower in the UPD population than in the DEL population. Overall, these findings suggest that the two populations differed in terms of cognitive strategies for making decisions and choosing which faces were similar.


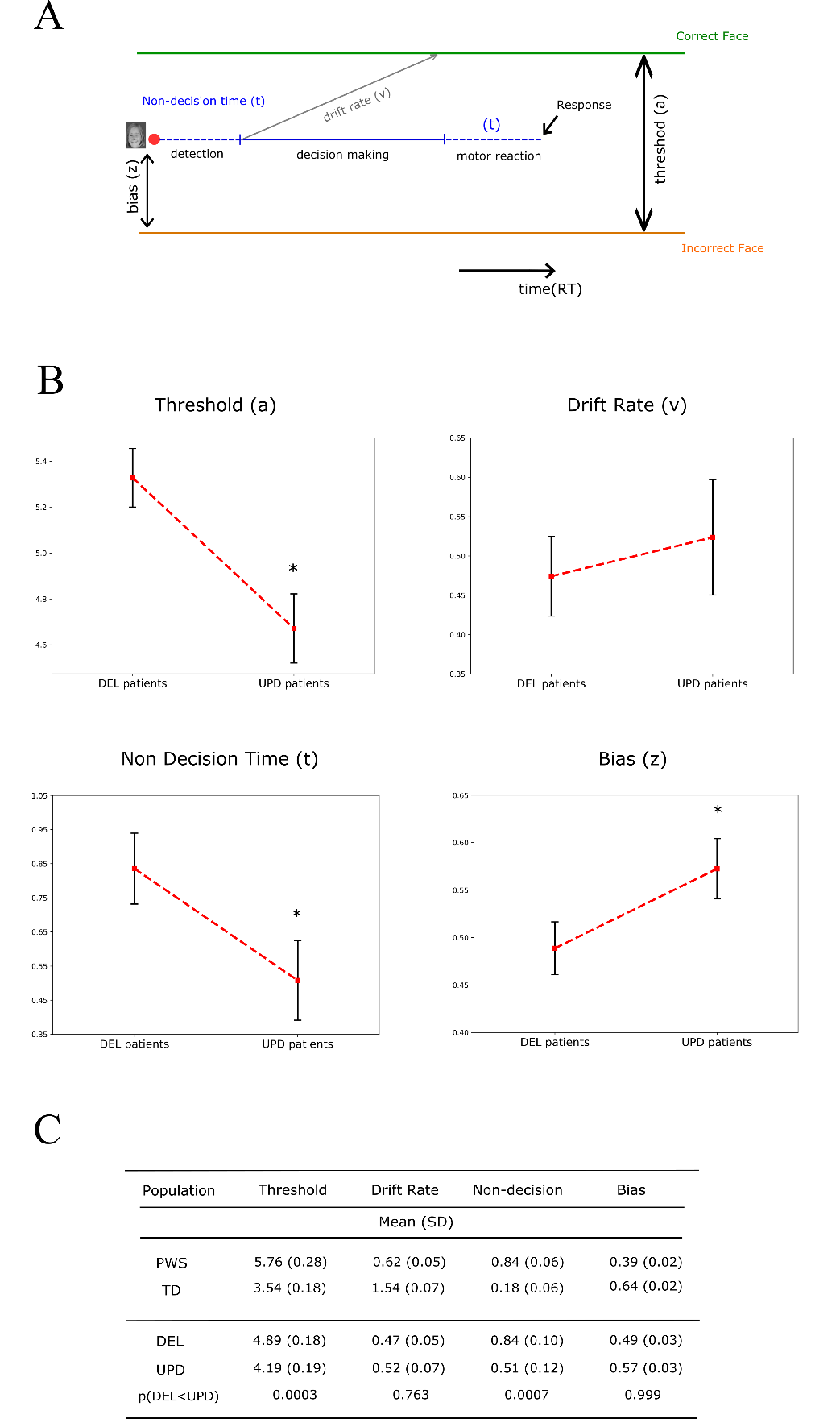


***Fig.S1:*** *Results of the Bayesian estimation of the drift diffusion model. A: Schematic view of* *the diffusion principle, adapted from Wiecki et al. (2013). To make a decision, participants had to integrate a certain amount of information, represented by the threshold (a), at a specific speed, represented by the drift rate (v). The nondecision time (t) was the time not involved in the decision making, reflecting instead the time taken to perform the motor control and to detect the stimulus. Finally, in this model we can calculate the bias (z) as an a priori decision based on the earlier presentation located between the two boundaries (right or wrong response). The total reaction time can be regarded as the sum of the four criteria specified above. B: Averages for each criterion (threshold, drift rate, nondecision time and bias) for the patients with DEL and UPD. Black bars represent the 95% confidence intervals, and asterisks indicate the statistical differences between patients with DEL and UPD. C: Table of all the results for each criterion, shown as means and standard deviations, and overlap between the two posterior distributions.*

**Comparison between groups**

We apply during our analysis a multiple comparison of each parameter between the 3 groups (DEL patients, UPD patients and TD controls). We did perform an intergroup analysis using the linear mixed effect model. For each parameter, at the exception of the fixation of the mouth area, we do not find any significant differences between groups probably because of a huge variability of behaviour in patients. Indeed, we observed that the UPD patients spend more time fixating the mouth region that the two other groups (DEL and TD).

|  | DEL patients | UPD patients | TD controls | DEL vs UPD | DEL vs TD | UPD vs TD |
| --- | --- | --- | --- | --- | --- | --- |
| Eye region | 54.4% | 30.4% | 59.4% | P<0.001 | P=0.44 | P<0.001 |
| Mouth region | 22.6% | 41.1% | 12.4% | P<0.001 | P=0.69 | P<0.001 |
| Nose region | 23% | 28.6% | 28.2% | P=0.52 | P=0.41 | P=0.99 |
| Sample_target | 41.6% | 39.2% | 39.6% | P=0.66 | P=0.59 | P=0.99 |
| Sample_distractor | 33.1% | 33.9% | 30.2% | P=0.89 | P=0.51 | P=0.39 |
| Target_distractor | 25.3% | 26.8% | 30.3% | P=0.91 | P=0.1 | P=0.47 |

***Table.S1:*** *Average values for each eye-tracking parameters and the corresponding p values obtained for comparison between the 3 groups.*

**Video presentation description**

Like Klin and colleague ^3^, we used short sequences from the movie *Who’s afraid of Virginia Woolf?*. These videos were chosen because of their socially rich content and the numerous interactions between the protagonists. No sequence lasted more than 40 seconds.

We divided the first sequence into two parts. Three characters interacted in the first one. A man on the right could be seen speaking to a woman on the left. The third character, a man, moved to the background to drink a glass of alcohol. We defined four AOIs for this sequence (faces of the characters and the background), for clarity’s sake, we only considered the three face AOIs. This first part lasted approximately 15 seconds. Immediately after, the sequence changed and the focus was on the two characters in the foreground. The woman interrupted her husband, who had previously been speaking, and she spoke alone for the rest of the sequence. We identified five AOIs: the face and body of the woman who was speaking, the face and body of the man, and the background.

The second sequence was chosen because it contained an interesting situation, during which the woman was seen eating a chicken leg (defined as an AOI in the analysis) while she spoke to another character. We chose this sequence because of the presence of a food stimulus‑important motivational information for patients with PWS. This sequence lasted 35 seconds. We defined five AOIs: the woman’s face, the woman’s body, the chicken leg, the man, and the background.

The third sequence was quite similar to the previous video, with only two characters interacting. At the beginning of the sequence, the man was sitting reading his newspaper and holding a cigarette. In the middle of the sequence, the woman took her husband's cigarette and asked him questions. The sequence lasted 38 seconds. We defined five AOIs: the man, the woman (face and body), the cigarette (exchanged between the man and the woman), and the background.

Participants were simply told they were free to watch the video as they liked. To ensure that participants were attentive, we asked them a question about the sequence they had just watched at the end of each video clip (e.g., “How many people did you see?”, “What was the woman eating?”, “What was exchanged during this video clip?”). Patients had a success rate of 91%, indicating that they were attentive to the video presentations.


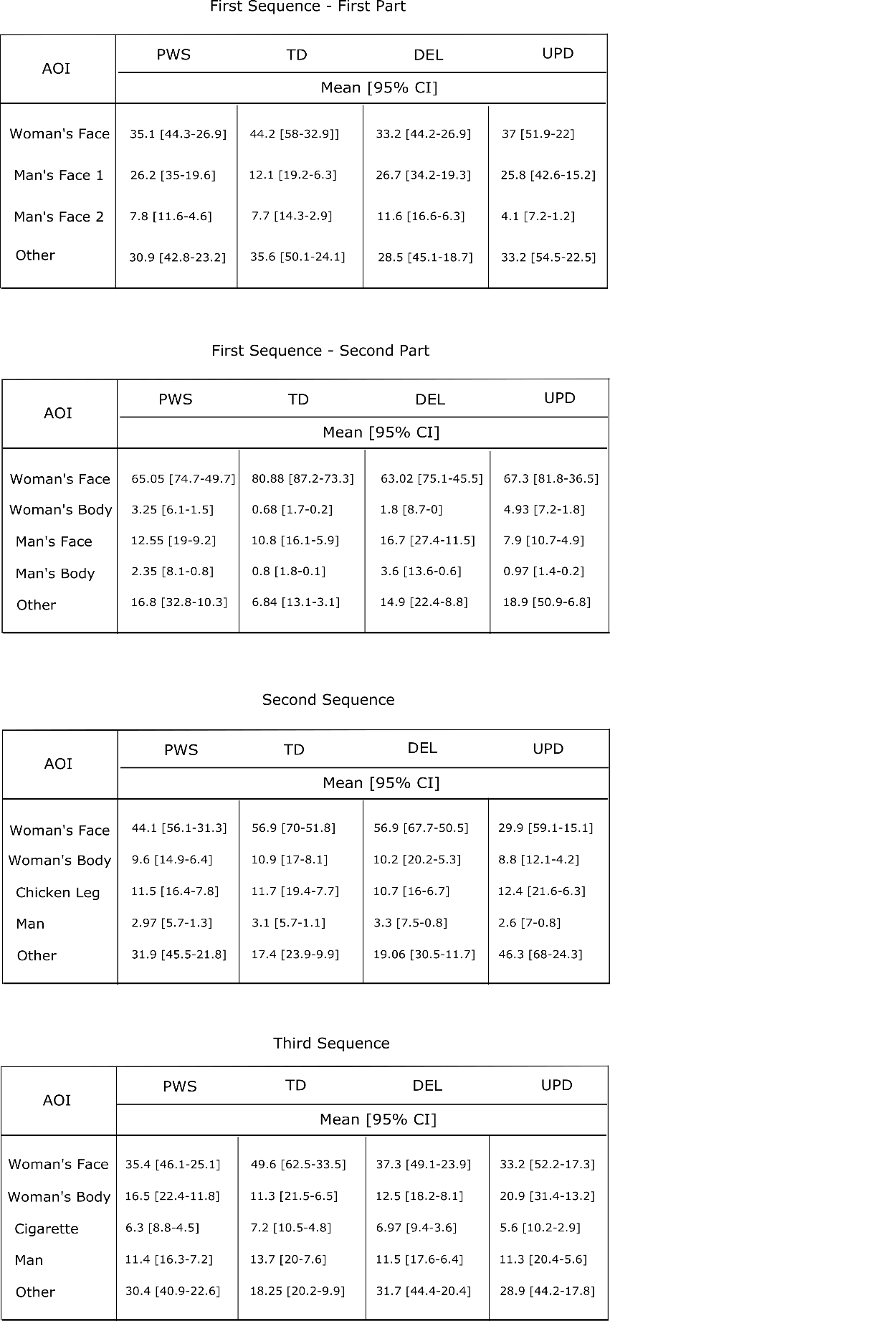


***Fig.S2:*** *Tables of the three sequences of the movie used in this study. In all the tables, we provide the percentage of fixation time for each AOI. The 95% confidence intervals are provided in parentheses.*

**Distinction between face and emotion face exploration**

This table illustrate the proportion of fixation on each area of interest (eye region, mouth region and nose region) and the p values inherent of the comparison between the two tasks. We show that the proportion of fixation on the eye region is lower during the emotion recognition task in DEL population. This difference is not significant for TD controls and UPD patients. During emotion recognition, DEL and UPD patients are more attracted by the mouth region than during face recognition task (respectively p=0.03 and p=0.01). The gaze proportion are not different for the nose region in the 3 groups.


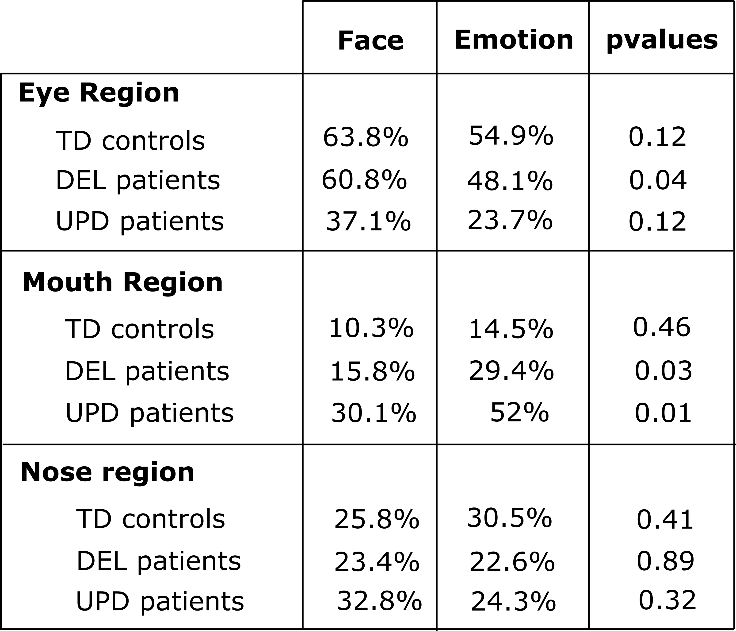


***Fig.S3****: Table of pvalues obtained by comparing the proportion of gaze fixation during face and emotion recognition task for the 3 different AOI and for each group.*

**References**

1. Ratcliff, R. & Rouder, J. N. Modeling Response Times for Two-Choice Decisions. *Psychol. Sci.* **9,** 347–356 (1998).

2. Wiecki, T. V., Sofer, I. & Frank, M. J. HDDM: Hierarchical Bayesian estimation of the Drift-Diffusion Model in Python. *Front. Neuroinformatics* **7,** (2013).

3. Klin, A., Jones, W., Schultz, R., Volkmar, F. & Cohen, D. Visual Fixation Patterns During Viewing of Naturalistic Social Situations as Predictors of Social Competence in Individuals With Autism. *Arch. Gen. Psychiatry* **59,** 809 (2002).
